# LncRNA DHRS4-AS1 ameliorates hepatocellular carcinoma by suppressing proliferation and promoting apoptosis via miR-522-3p/SOCS5 axis

**DOI:** 10.1080/21655979.2021.1994719

**Published:** 2021-11-29

**Authors:** Yongping Zhou, Kuan Li, Xuexia Zou, Zhiyuan Hua, Hao Wang, Wuyang Bian, Hong Wang, Fangming Chen, Tu Dai

**Affiliations:** a Department of Hepatobiliary, Wuxi No.2 People’s Hospital, Affiliated Wuxi Clinical College of Nantong University, Wuxi, Jiangsu, China; b Department of Hepatobiliary Surgery, Kunshan Hospital of Traditional Chinese Medicine, Kunshan, Jiangsu, China; c Department of Operation Room, Wuxi No.2 People’s Hospital, Affiliated Wuxi Clinical College of Nantong University, Wuxi, Jiangsu, China; d Department of Imagine, Wuxi No.2 People’s Hospital, Affiliated Wuxi Clinical College of Nantong University, Wuxi, Jiangsu, China

**Keywords:** Hepatocellular carcinoma, DHRS4-AS1, miR-522-3p, SOCS5, proliferation

## Abstract

Recent years have seen much effect in revealing the pathological association between lncRNA and HCC. Herein, we identified lncRNA DHRS4-AS1 as a potential tumor suppressor in HCC. Firstly, it was discovered that DHRS4-AS1 was significantly down-regulated in HCC tissues compared to normal tissues based on the database TCGA. It was also detected in a lower-than-usual expression quantity in HCC tissues we collected and HCC cell lines. Kaplan-Meier survival analysis revealed that high expression of DHRS4-AS1 contributed to higher overall survival rate of HCC patients.DHRS4-AS1 expression was significantly correlated to tumor size (*P* = 0.02) and TNM stage (*P* = 0.045). CCK-8, BrdU and colony-forming assays collectively demonstrated that overexpression of DHRS4-AS1 significantly restrained HCC cell proliferation. In vivo xenograft animal experiment showed that DHRS4-AS1 could efficiently preclude the tumor growth of HCC. Further investigation performed using flow cytometry and western blot showed that DHRS4-AS1 exerted its effects by accelerating cell apoptosis and capturing cell cycle in G0/G1 phase. Our study subsequently lucubrated that miR-522-3p was a negative target of DHRS4-AS1. Increased expression level of miR-522-3p was examined in HCC tissues and cell lines. Similarly, miR-522-3p mimics could reverse the inhibitory effect on HCC brought by DHRS4-AS1. SOCS5 was then discovered as a down-stream target of miR-522-3p, which suggested that SOCS5 participated in DHRS4-AS1/miR-522-3p axis to collectively mediate the development of HCC. Our study provides lncRNA DHRS4-AS1/miR-522-3p/SOCS5 axis as a novel target for HCC therapeutic strategy with potentiality.

## 1. Introduction

Hepatocellular carcinoma (HCC) has been considered as the most common primary malignant cancer among hepatic cancer with a proportion of around 70 ~ 90%, which is also ranked as the sixth largest solid tumor worldwide and the fourth leading cause of high mortality [1–3]. Based on the previous reported epidemiological and experimental studies, the pathogeny and rapid occurrence of HCC have close correlation with multifarious external environmental factors, viral diseases, and medication. So far, HCC is characterized by high heterogeneity, environmental or genetic susceptibility, morphological diversity and signal network disorder according to primitive studies [4–6]. The 5-year age standardized relative survival rate of HCC only reached 12.1% in China because of the high difficulty of early diagnosis [7], and most of the HCC patients have already developed to terminal stage when receiving a consultation [8]. Moreover, the rapid development, high degree of malignance, and poor curative effect of chemotherapy for advanced hepatocellular carcinoma are also listed as the sophisticated reasons for the low survival rate of HCC. Nevertheless, the exact mechanism of HCC occurrence and development still not been fully elucidated [9]. Therefore, exploration for the exact molecular mechanism of HCC possibly provides novel therapeutic strategy for early diagnosis.

Long non coding RNA (lncRNA) is a class of RNA transcripts without proteins coding capability, of which the length is usually over 200 nt [10]. It is reported that lncRNA is a sophisticated participant in modulating multifarious biological procedures including cell growth, apoptosis, autophagy, migration, and invasion [11]. In particular, the ectopic expression of lncRNA might has some pathological correlation with carcinogenesis, tumor development, metastasis, recurrence and chemoresistance [12–15]. Recent years have seen much effect in revealing the pathological association between lncRNA and hepatocellular carcinoma, of which lncRNA is considered as a pivotal factor that partakes in the occurrence, development and metastasis of HCC via the interaction with DNA, RNA and related proteins [16]. According to the preliminary literature research, lncRNA DHRS4-AS1 functions as a tumor suppressor in a majority of cancers, including glioma, clear cell renal cell carcinoma, and neuroblastoma [17–19]. However, there is little understanding of the specific role of DHRS4-AS1 in HCC.

Among the abundant researches of lncRNA in tumorigenesis, lncRNA further mediates the expression level of targeted microRNA (enhance or inhibit) and thereby controlling the development of cancer [20,21]. Current studies of miR-522-3p have illuminated it as an oncogene of various types of tumors. Shuai et al elucidated in their study that miR-522-3p promotes the proliferation of colorectal cancer by negatively regulating the expression of bloom syndrome protein (BLM) [22]. Additionally, research done by Chen et al disclosed that miR-522-3p accelerates the cell growth of osteosarcoma via mediating glucose uptake and the quantity of GLUT1 [23]. Yet the specific role of miR-522-3p in hepatocellular carcinoma has never been discussed. In present study, we aim at disclosing the regulatory pathway of DHRS4-AS1 and consequently provided a novel direction in investigating curative method for HCC with potentiality.

Suppressor cytokine signaling-5 (SOCS5) is widely investigated for its regulatory function in carcinogenesis and tumor development [24]. Researchers have reached a consensus that SOCS5 functions as a tumor suppressor in various types of carcinomas. According to the study of Zhang et al, SOCS5 was mediated by miR-302a-3p and thereby ameliorated the migration of pancreatic cancer [25]. Similarly, another research study revealed that SOCS5 partakes in impairing the invasive and metastatic capacity of pancreatic cancer via JAK/STAT3 pathway [26]. Recent years have seen much effect on the specific function of SOCS5 in cancers, including liver cancer [27,28], glioma [29], chronic lymphocytic leukemia [30], and breast cancer [31]. Yet the exact biological function of SOCS5 in HCC has not been elaborated.

Here, this study aimed at disclosing the specific function of lncRNA DHRS4-AS1 in HCC and also investigated the underlying mechanism of lncRNA DHRS4-AS1 in promoting HCC progression, so as to provide a novel method and strategy for early diagnosis and regimen of HCC with potentiality.

## 2. Materials and methods

### 2.1. Bioinformatics prediction and survival rate analyses through TCGA database

TCGA (https://www.cancer.gov/) database was an online sequencing tool that assistant with the mRNA expressed prediction of DHRS4-AS1 in hepatocellular carcinoma tissues and normal para-carcinoma tissues.

### 2.2. Acquisition of HCC tissues samples

A total of 60 pairs of hepatocellular carcinoma tissues and adjacent normal tissues were resected from HCC patients who have received surgical therapy in Wuxi No.2 People’s Hospital. The tumor tissues and matched adjacent tissues of all patients were freshly collected, and the adjacent tissues were more than 2 cm from the edge of the tumor, all patients have not received any anti-tumor treatment prior to surgery.

Meanwhile, the overall survival rate of HCC patients with surgical treatment was analyzed to give the prediction of HCC patients with different expression quantity of DHRS4-AS1 via Kaplan-Meier survival estimators, and the comparison of the consequence of two groups was carried out using log-rank tests. All the participants have signed the informed consent according to the requirement of the local institutional review board. The extracted tissues were subsequently frozen using liquid nitrogen and hoarded under −80°C till experiments.

### 2.3. Cell culture and transfection

Normal human hepatic cell line L02, and human hepatocellular carcinoma cell lines including Hep3B, YY-8103, Focus, HCCLM3 and Huh7 (American Type Culture Collection, Manassas, VA, USA) were incubated in DMEM medium (Gibco, USA) with 10% FBS (Gibco, USA) at a condition of 37°C and 5% CO_2._ Then seeded HCCLM3 and YY-8103 cells which were in logarithmic growth period in six-well plate according to 2 × 10^5^ cells per well. Applying transfection assays according to the instructions of Lipofectamine^TM^ 2000. The overexpression plasmid (DHRS4-AS1 ov group)/control group (NC group), miR-522-3p mimics, miR-522-3p inhibitor/negative sequence (inhibitor NC group), and small interfering RNA of SOCS5 (si-SOCS5) were transfected into HCCLM3 cell line, respectively. The DHRS4-AS1 expression suppressor plasmid (si-DHRS4-AS1 group)/negative control plasmid (si-NC group) was transfected into YY-8103 cell line. All of these plasmids were purchased from GenePharma (Shanghai, China) and the sequences have been provided in Table 1.

**Table 1.** The sequences used in this study

Name	Sequences
DHRS4-AS1 forward primer	5ʹ‑GGAGGCTGAGGCAGGAGAAT‑3’
DHRS4-AS1 reverse primer	5ʹ‑GCTAGTCTGGTCACCTCTGGAT‑3’
miR-522-3p forward primer	5ʹ‑CGCGAAAATGGTTCCCTTTA‑3’
miR-522-3p reverse primer	5ʹ‑AGTGCAGGGTCCGAGGTATT‑3’
SOCS5 forward primer	5ʹ‑GTGCCACAGAAATCCCTCAAA‑3’
SOCS5 reverse primer	5ʹ‑TCTCTTCGTGCAAGTCTTGTTC-3’
U6 forward primer	5ʹ-CTCGCTTCGGCAGCACA‐3’
U6 reverse primer	5ʹ-GGATGGTGATGGTTTGGTAG‐3’
GAPDH forward primer	5ʹ‐CTCGCTTCGGCAGCACA‐3’
GAPDH reverse primer	5ʹ-AACGCTTCACGAATTTGCGT‐3’
sh-DHRS4-AS1	5ʹ-GAACTGTACTCTTACTCGAGTAAGAGTACAGTTCTGTC CTTTTTT‐3’
miR-522-3p mimics	5ʹ-TCTCAGGCTGTGTCCCTCTAGAGGGAAGCGCTTTCTG TTGTCTGAAAGAAAAGAAAATGGTTCCCTTTAGAGTGTTACGCTTTGAGA‐3’
miR-522-3p inhibitor	5ʹ-CTCTAGAGGGAAGCGCTTTCTG‐3’
si-SOCS5	5ʹ-GATAATGATTCTTGTGTTACTCGAGTAACACAAGAATC ATTATCTTTTTT‐3’

### 2.4. Total RNA extraction and qRT-PCR

Human HCC tissues and adjacent normal tissues were grant for further RNA extraction. Tissues homogenate as well as normal hepatic cells (L02) and HCC cells (Hep3B, YY-8103, Focus, HCCLM3 and Huh7) were added 1 ml of Trizol reagent (Invitrogen, Carlsbad, CA, USA) to lyse cells. The cDNA synthesis was conducted using reverse transcription reagents (Thermo Scientific, Waltham, MA, USA). Chloroform was then added to the mixture at ambient temperature, which was followed by the addition of isopropanol. Ultimately, the suspension was centrifugated to obtain RNA from the sediments. The expression of DHRS4-AS1 and SOCS5 were determined by qRT-PCR by utilizing the PrimeScript RT reagent Kit and SYBR Prime Script RT-PCR Kits (TaKaRa Bio, Inc., Shiga, Japan) according to the manufactures’ guidance, and GAPDH as the internal control. The expression of miR-522-3p was measured using TaqMan MicroRNA Assays and U6 as the internal control. 2^−ΔΔct^ were used as the calculating method for the results data. The primer sequences have been provided in Table 1.

### 2.5. Western blot

Cells in the logarithmic period were collected and lysed using lysis buffer at freezing point for 30 min. BCA Kit (Bio-Rad Laboratories USA) was adopted to quantify the total proteins. Proteins solution was boiled for denaturation after the addition of loading buffer. After the proteins separation on SDS-PAGE, proteins were transferred onto polyvinyldifluoride membranes (Thermo Scientific, Waltham, MA, USA). The diluent (1:1000) of primary antibodies, involving Bcl-2 (ab32124, Abcam), Bcl-xl (ab32370, Abcam), Bax (ab32503, Abcam), Cyclin-D1 (ab16663, Abcam), SOCS5 (ab97283, Abcam) and GAPDH (ab8245, Abcam), were added to immerse the membranes overnight with gentle rocking. After washing with TBST, the membranes were incubated with the appropriate horseradish peroxidase-conjugated secondary antibodies (SA00001-1 or SA00001-2, Proteintech) diluted with 1:2000 for 2 h at 37°C. After extensive washing with TBST, observation of proteins strap was conducted using chemiluminescence reagent (Thermo Scientific, Waltham, MA, USA).

### 2.6. Cell counting Kit-8 (CCK8) assay

HCC cells in the logarithmic period were seeded onto a 96-well plate and each well has a density of 10^3^ cells. 10 μL of CCK8 reagent (Dojindo, Tokyo, Honshu, Japan) was added at 24 h, 48 h, 72 h and 96 h. Ultimately, after 2 h of incubation of CCK8 reagent, the absorbance (OD450) was examined by a microplate reader (Thermo-Fisher Scientific).

### 2.7. Brdu cell proliferation assay

HCC cells in the logarithmic period were added with 10 μM of BrdU (Thermo Fisher Scientific) and incubated for 48 h. Colchicine (Sigma-Aldrich, USA) was then added to establish a concentration of 0.1 μM. HCC cells were subsequently collected and given a treatment of Giemsa solution (Sigma-Aldrich, USA) after 48 h of incubation. The stained cells were photographed and scrutinized by a fluorescence microscope.

### 2.8. Colony-forming assay

HCC cells were then seeded in six-well plate with a density of 500 cells per well. After 2-week of incubation, cells were fixed with 4% paraformaldehyde and stained using 1% crystal violet. Ultimately, the colonies quantity was counted via microscope to measure HCC cells proliferative capacity.

### 2.9. Dual-luciferase assay

The wild-type (WT) or mutated (Mut) of synthetic DHRS4-AS1 and SOCS5 (Sangon, Shanghai, China) were inserted into the pGL3 vector (Promega Corporation, Madison, USA) to construct the reporter plasmids. MiR-522-3p mimic and negative control (miR-NC) were co-transfected with the reporter plasmids by Lipofectamine 2000. Dual-Luciferase Reporter Gene Assay System was performed to determine the Renilla and Firefly luciferase activities.

### 2.10. Cell cycle and apoptosis measurement by flow cytometry

For cell apoptosis assay, 5 × 10^5^ HCC cells were suspended in 100 μL buffer and an addition of 5 μL annexin V and 5 μL 7-AAD was given to the cells. Cells were then incubated in dark at ambient temperature for 15 min. Thereafter, 400 μL of combined buffer was added, and cells were placed on ice (keep in a dark environment), and were determined through flow cytometry within 60 min.

For cell cycle assay, after transferring cells into 12-well plate and incubating for 24 h, 75% pre-cold ethanol was adopted to fix cells, following by incubation at 4°C for 7 h. Subsequently, all fixed cells were rinsed with pre-cool PBS three times. Propidium iodide (PI) was used to stain cell at ambient temperature for 30 min, the whole operation was exerted in a dark environment. Finally, cell cycle distribution was analyzed through flow cytometry.

### 2.11. Animal study

We used sixteen 6–8 week-old male BALB/C nude mice, weighting 18 g – 20 g, to establish animal models, that were purchased from Cavens (Changzhou, China) and raised in aseptic environment. The conducted animal study was permitted and strictly adheres to the approval of the Care and Use of Laboratory Animals. The method was followed the previous study [32]. HCC cells were prepared and injected into BALB/c nude mice for the tumor model construction. Every 3 days, the tumor volume was gauged and recorded. After 21-day of feeding, the mice were executed for the measurement of tumor weight.

### 2.12. Statistical analyses

SPSS 20.0 software was used for statistical analysis, and the measurement data were expressed by mean ± standard deviation (x ± s). T-test was used for comparison between the two groups; comparison between groups was evaluated through one-way analyses of variance. Categorical data were assessed using a chi-square test. Kaplan-Meier and log-rank analysis were used for survival analysis. P < 0.05 meant the difference was statistically significant.

## 3. Results

### 3.1. LncRNA DHRS4-AS1 demonstrated descent abundance in HCC tissues and cell lines

**Figure 1.**
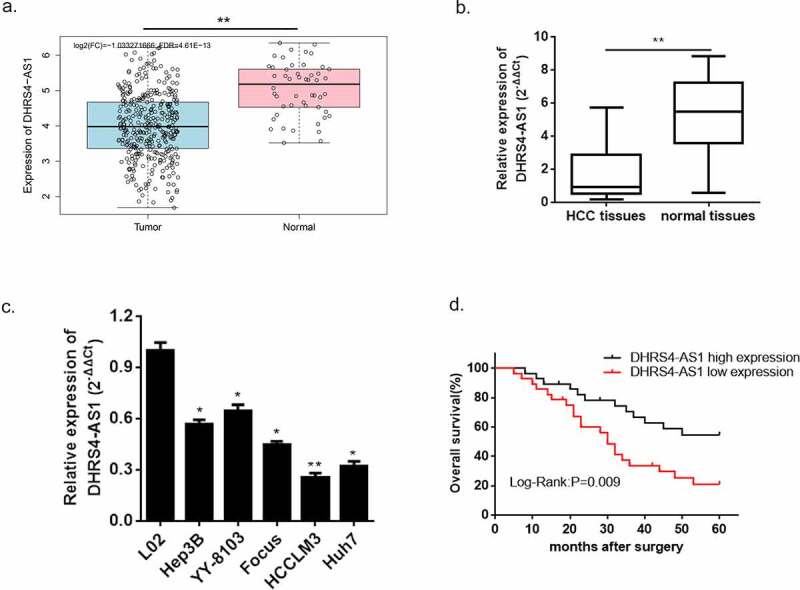
LncRNA DHRS4-AS1 was in low expression in HCC tissues and cell lines. (a) DHRS4-AS1 was predicted in descent expression level in HCC via TCGA database. (b) QRT-PCR exhibited significant reduction of DHRS4-AS1 expression in HCC tissues. (c) The level of DHRS4-AS1 in HCC cell lines. (d) The overall survival rate of HCC patients with different expression level of DHRS4-AS1. N = 3, *p < 0.05, **p < 0.01

Based on our preliminary literature study, LncRNA DHRS4-AS1 functions as a tumor suppressor via regulating the biological process of multifarious types of cancer [18]. Nevertheless, there is little discussion about the exact regulatory effect of DHRS4-AS1 in HCC. Herein, we conducted a bioinformatics prediction using TCGA database, and the predicted consequence showed that the expression of DHRS4-AS1 was significantly lower in HCC tissues versus normal hepatic tissues (Figure 1(a)). To further verify this prediction, we collected a total of 60 pairs of HCC tissues and adjacent normal tissues from HCC patients, and performed an estimation of DHRS4-AS1 via qRT-PCR. The result shown in (Figure 1(b)) supported the significant reduction of DHRS4-AS1 abundance in HCC tissues when compared with the adjacent normal tissues (p < 0.001). Similarly, the expression level of DHRS4-AS1 was also observed in hepatic normal cell line (L02) and HCC cell lines (Hep3B, YY-8103, Focus, HCCLM3 and Huh7). The results also displayed significantly reduced mRNA content of DHRS4-AS1 in HCC cell lines (*p < 0.05, **p < 0.01) when compared with hepatic normal cell line. Next, the overall survival rate of patients with DHRS4-AS1 high expression or low expression was evaluated. Kaplan-Meier survival analyses described that HCC patient with high expression of DHRS4-AS1 own an increased overall survival rate versus low expression (Figure 1(d)). Furthermore, we studied the relationship between DHRS4-AS1 expression and the clinicopathologic features of HCC patients. As shown in Table 2, DHRS4-AS1 expression was significantly correlated to tumor size (*P* = 0.02) and TNM stage (*P* = 0.045). However, it was not related to age, gender, liver cirrhosis, HBsAg status, AFP, tumor multiplicity or Edmondson grade. The consequences displayed above suggested a hypothesis that up-regulation of DHRS4-AS1 could possibly ameliorate the course of HCC.

**Table 2.** Correlation of DHRS4-AS1 expression with clinicopathological characteristics in HCC patients

characteristic	case	DHRS4-AS1 expression		*P* value
		low	high	
All case	60	30	30	
Age (years)				0.436
<60	27	12	15	
≥60	33	18	15	
Gender				0.787
Female	21	11	10	
Male	39	19	20	
Liver cirrhosis				0.197
No	12	4	8	
Yes	48	26	22	
HBsAg status				0.592
Negative	22	12	10	
Positive	38	18	20	
AFP (ng/ml)				0.405
≤ 20	19	11	8	
> 20	41	19	22	
Tumor size (cm)				0.020*
≤ 5	44	18	26	
> 5	16	12	4	
Tumor multiplicity				0.426
Single	37	20	17	
Multiple	23	10	13	
Edmondson grade				0.436
I–II	33	15	18	
III–IV	27	15	12	
TNM stage				0.045*
I–II	43	18	25	
III–IV	17	12	5

### 3.2. LncRNA DHRS4-AS1 restricted HCC cells proliferation and promoted cell apoptosis

**Figure 2.**
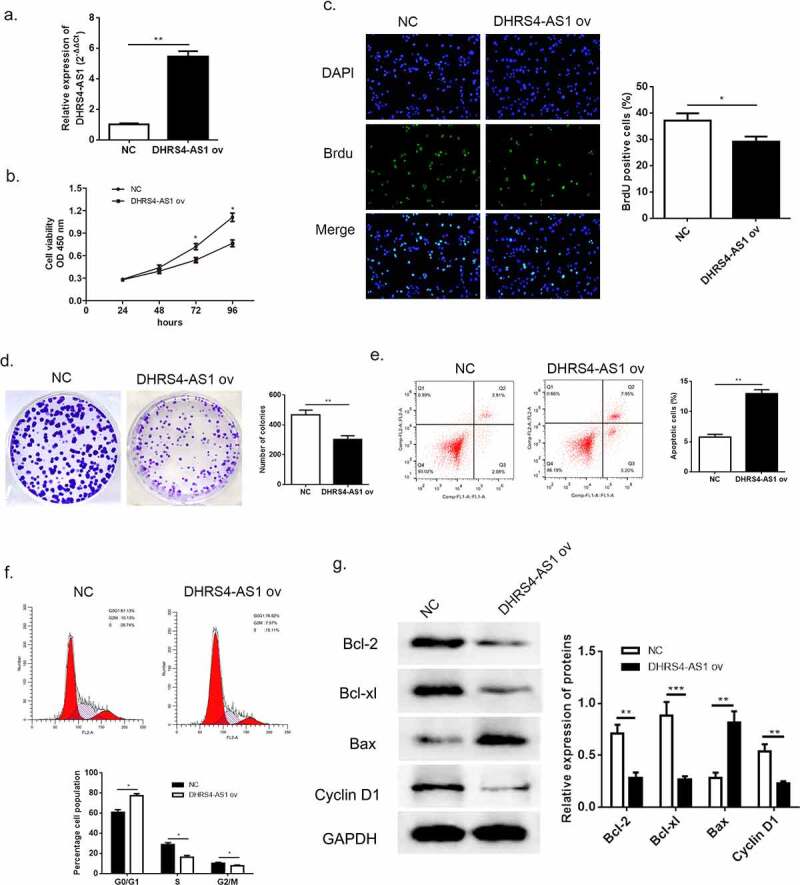
Overexpression of DHRS4-AS1 restricted HCC cell growth in vitro. (a) The expression level of DHRS4-AS1 determined by qRT-PCR. (b) Cell proliferation of HCC with DHRS4-AS1 overexpression that estimated by CCK-8. (c) The result of BrdU assay. (d) Colony-forming assay demonstrated decreased cell proliferation in DHRS4-AS1 up-regulated HCC cells. (e-f) Flow cytometry showed that overexpression of DHRS4-AS1 resulted in potentiated cell apoptosis, and cell cycle was captured at G0/G1 phase. (g) The expression level and quantitative analysis of apoptosis related proteins and cell cycle related protein detected via western blot. N = 3, *p < 0.05

**Figure 3.**
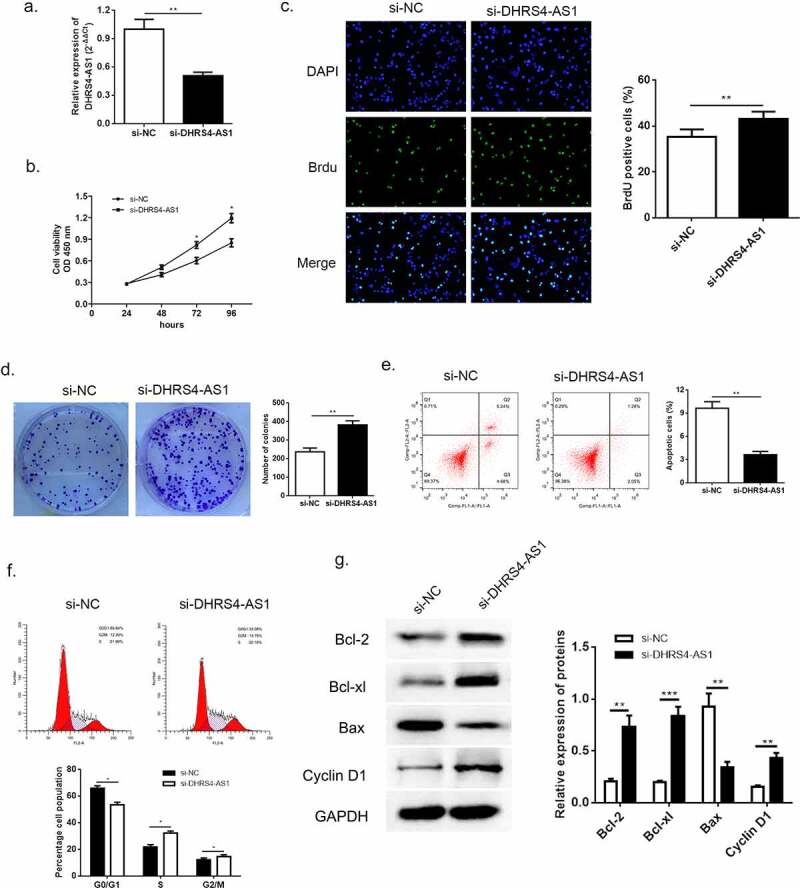
Knockdown of DHRS4-AS1 accelerated HCC cell growth in vitro. (a) The expression level of DHRS4-AS1 determined by qRT-PCR. (b) Cell growth of HCC with DHRS4-AS1 down-regulation that determined through CCK-8. (c) The result of BrdU assay. (d) Colony-forming assay demonstrated enhanced cell proliferation in DHRS4-AS1 down-regulated HCC cells. (e-f) Flow cytometry showed that silencing of DHRS4-AS1 resulted in alleviated cell apoptosis, and cell cycle that captured at G0/G1 phase dropped. (g) The expression level and quantitative analysis of apoptosis and cell cycle related protein detected via western blot. N = 3, *p < 0.05

According to the previous evidence of descent DHRS4-AS1 in HCC tissues and cell lines, we proposed an assumption that lncRNA DHRS4-AS1 might validate in inhibiting the development of HCC. As described in (Figure 1(c)), HCCLM3 cell line presented the lowest DHRS4-AS1 quantity, while YY8103 cell line had the highest. Hence, DHRS4-AS1 was designed to up-regulate in HCCLM3 cell line (Figure 2(a), p < 0.05), and down-regulate in YY-8103 cell line (Figure 3(a), p < 0.01), respectively. Subsequently, the alteration of cell growth was scrutinized using CCK-8 assay, Brdu assay and colony-formation assay. CCK-8 assay showed that cells escalated slower than NC group when there was overexpression of DHRS4-AS1 (DHRS4-AS1 ov) in HCCLM3 (Figure 2(b)), while YY-8103 with DHRS4-AS1 knockdown (si-DHRS4-AS1) presented rapid cell growth (Figure 3(b)). Simultaneously, BrdU assay and colony-forming assay displayed that up-regulation of DHRS4-AS1 in HCCLM3 manifested significant lower ratio of BrdU positive cells (Figure 2(c), p < 0.05), and impaired colony-forming ability (Figure 2(d), p < 0.01). Conversely, YY-8103 with DHRS4-AS1 knockdown displayed higher BrdU positive cell ratio (Figure 3(c), p < 0.01), and potentiated cell growth capacity (Figure 3(d), p < 0.01). The effect of DHRS4-AS1 on cell cycle and apoptosis was investigated using flow cytometry. Results shown in (Figures 2(e–f)) suggested that overexpression of DHRS4-AS1 promoted HCCLM3 cells apoptosis (p < 0.01) and captured cell cycle to a retention of G0/G1 phase (p < 0.05). While DHRS4-AS1 silencing in YY-8103 cell line provided an opposite phenomenon (Figures 3(e–f)) that the apoptosis rate dropped below the control group (p < 0.01) and the ratio of G0/G1 phase reduced compared to the control group (p < 0.05). Western blot was carried out to examine the abundance of apoptosis-related proteins (Bcl-2, Bcl-xl and Bax) and cell cycle related protein Cyclin-D1. Up-regulation of DHRS4-AS1 in HCC cells demonstrated significant reduction of Bcl-2, Bcl-xl and Cyclin-D1, but the abundance of Bax increased (Figure 2(g)). Instead, the expression of Bcl-2, Bcl-xl and Cyclin-D1 in DHRS4-AS1 silencing cells rose while Bax decreased (Figure 3(g)). Conclusively, our data illuminated that the proliferation suppressed function of lncRNA DHRS4-AS1 was validated in vitro.

### 3.3. LncRNA DHRS4-AS1 attenuated tumor growth in vivo

**Figure 4.**
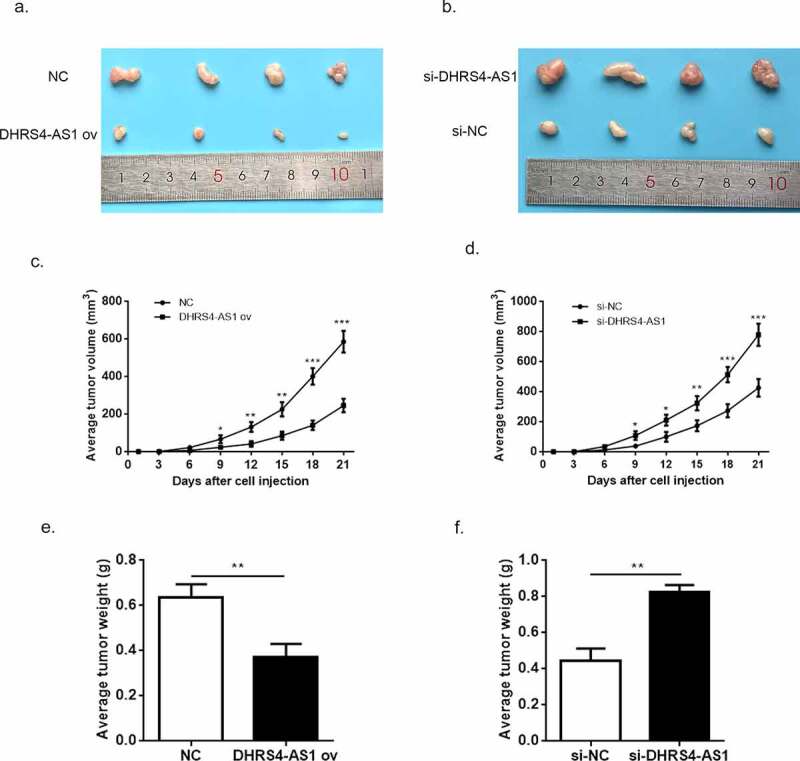
The tumor suppressed effect of DHRS4-AS1 in vivo. (a-b) Tumor size of HCC after 21-day of treatment. (c-d) The average tumor volume of HCC during 21-day of treatment. (e-f) The average tumor weight of HCC during 21-day of treatment. N = 4, ***p < 0.001

Although the tumor suppressed function of DHRS4-AS1 has been proved in vitro, the exact effects in vivo still need to be discussed. Herein, we constructed HCC xenograft model using BALB/c nude mice. The HCC cells transfected with NC, DHRS4-AS1, si-NC and si-DHRS4-AS1 were injected into mice to observe the tumor proliferation. The average tumor volume in DHRS4-AS1 ov group escalated slower than NC group (Figure 4c); while in si-DHRS4-AS1 group, tumor grown in a rapid pace (Figure 4d). After 21-day of examination, mice were executed and resected the tumor tissues for comparison. Depicted in (Figure 4(a,b)) proved that up-regulation of DHRS4-AS1 has smaller tumor size when compared to control, while knockdown of DHRS4-AS1 showed bigger size than si-NC with significant difference. Similarly, the average tumor weight in DHRS4-AS1 ov group presented significant reduction (Figure 4(e), p < 0.01) while increase of tumor weight was observed in si-DHRS4-AS1 group (figure 4(f), p < 0.01). These consequences collectively elucidated that DHRS4-AS1 was capable to reduce HCC tumor grow in vivo.

### 3.4. miR-522-3p was identified as a negatively regulator target of DHRS4-AS1 and reversed the effects of DHRS4-AS1

**Figure 5.**
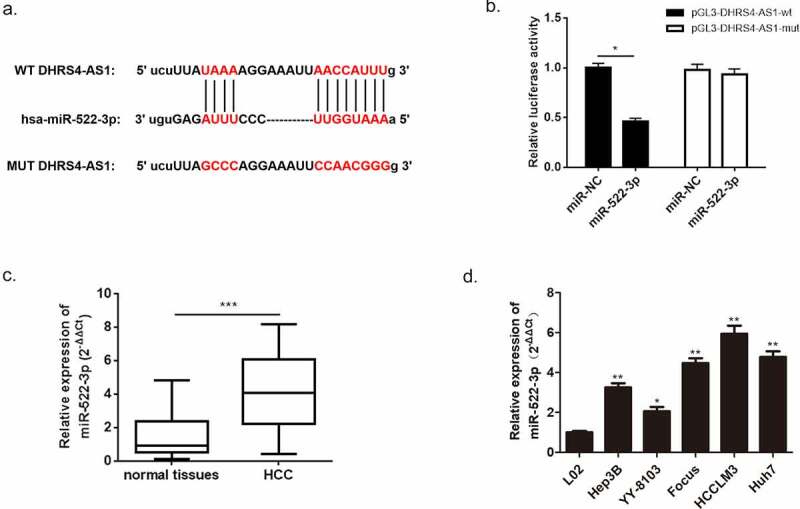
miR-522-3p was identified as a target gene of DHRS4-AS1. (a) The bioinformatics prediction biding site. (b) The result of dual-luciferase assay. (c) miR-522-3p was overexpressed in HCC tissues. (d) QRT-PCR detected significant high expression of miR-522-3p in HCC cell lines. N = 3, *p < 0.05, **p < 0.01

Further investigation of the molecular mechanism of DHRS4-AS1 was conducted using miRanda database. We then performed a bioinformatics prediction and discovered an overlapping sequence between miR-522-3p and DHRS4-AS1, which suggested that there might be an interaction between these two factors. To confirm this speculation, dual-luciferase assay was carried out and proved the indeed existence of interaction between miR-522-3p and DHRS4-AS1 (Figure 5(b), p < 0.05). Subsequently, the expression level of miR-522-3p in HCC patient’s tissues and HCC cell lines were measured using qRT-PCR to further understand the correlation between miR-522-3p and HCC. Results manifested overexpression of miR-522-3p in HCC tissues versus normal tissues (Figure 5(c), p < 0.001). Also, up-regulation of miR-522-3p showed in HCC cell lines when compared with normal hepatic cell (Figure 5(d), *p < 0.05, **p < 0.01). Furthermore, we studied the relationship between miR-522-3p expression and the clinicopathologic features of HCC patients. As shown in Table 3, miR-522-3p expression was significantly correlated to AFP (*P* = 0.012), tumor size (*P* = 0.004), tumor multiplicity (*P* = 0.017) and TNM stage (*P* = 0.002). However, it was not related to age, gender, liver cirrhosis, HBsAg status or Edmondson grade.

**Table 3.** Correlation of miR-522-3p level with clinicopathological characteristics in HCC patients

characteristic	case	miR-522-3p level		*P* value
		low	high	
All case	60	30	30	
Age (years)				0.519
<60	27	14	13	
≥60	33	16	17	
Gender				0.417
Female	21	9	12	
Male	39	21	18	
Liver cirrhosis				0.519
No	12	7	5	
Yes	48	23	25	
HBsAg status				0.284
Negative	22	13	9	
Positive	38	17	21	
AFP (ng/ml)				0.012*
≤ 20	19	14	5	
> 20	41	16	25	
Tumor size (cm)				0.004**
≤ 5	44	27	17	
> 5	16	3	13	
Tumor multiplicity				0.017*
Single	37	23	14	
Multiple	23	7	16	
Edmondson grade				0.194
I–II	33	14	19	
III–IV	27	16	11	
TNM stage				0.002**
I–II	43	27	16	
III–IV	17	3	14

**Figure 6.**
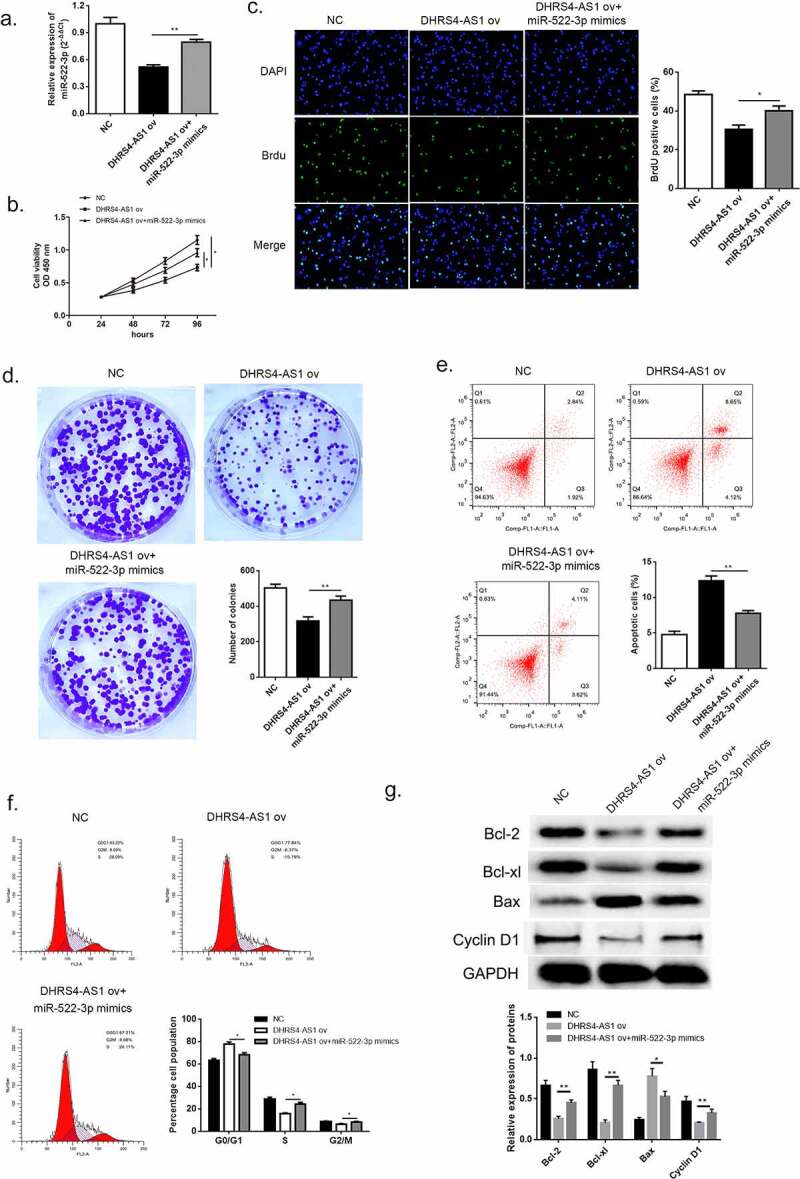
miR-522-3p reversed the inhibitory effects brought by DHRS4-AS1. (a) QRT-PCR determined the expression level of miR-522-3p. (b) CCK-8 assay showed that miR-522-3p promoted cell proliferation in DHRS4-AS1 and miR-522-3p mimics co-treated group versus solo DHRS4-AS1 ov treated group. (c) The result of BrdU assay. (d) Colony-forming experiment showed that miR-522-3p reversed the cell suppressed effect brought by DHRS4-AS1. (e-f) Cell apoptosis rate and cell cycle examined by flow cytometry. (g) The level and quantitative analysis of apoptosis related proteins’ and cell cycle related protein’s abundance detected via western blot. N = 3, *p < 0.05, **p < 0.01

From the consequences of qRT-PCR we suggested a hypothesis that miR-522-3p might serve as an oncogene and thereby potentiate the progress of HCC. Lucubration of the pathological function of miR-522-3p in HCC was conducted by co-transfecting miR-522-3p mimic and DHRS4-AS1 into HCC cells. QRT-PCR certified the success transfection of miR-522-3p mimics by comparing the mRNA content between DHRS4-AS1 ov group and DHRS4-AS1 ov + miR-522-3p mimics co-transfected group (Figure 6(a), p < 0.01). Thereafter, CCK-8 evaluation, BrdU assay and colony-forming experiment were conducted to explore the alteration of HCC cells. As described in (Figure 6(b)), up-regulation using miR-522-3p mimics enhanced the cell viability which was impaired by DHRS4-AS1 (Figure 6(b), p < 0.05). Meanwhile, higher proportion of BrdU positive cells showed in DHRS4-AS1 ov + miR-522-3p mimics co-transfected group (Figure 6(c), p < 0.05 compared to DHRS4-AS1 ov group). When giving miR-522-3p mimics to DHRS4-AS1 ov cells, cell proliferative ability raised, which probably illustrated that miR-522-3p was capable to reverse the effects brought by DHRS4-AS1. Mechanism exploration of miR-522-3p was performed via flow cytometry and western blot. The results of flow cytometry demonstrated that the apoptosis enhanced by DHRS4-AS1 was subsequently inhibited when giving miR-522-3p transfection (Figure 6(e), p < 0.01). Furthermore, overexpression of miR-522-3p could reduce the ratio of G0/G1 phase which potentiated by DHRS4-AS1 (figure 6(f), p < 0.05). The result of western blot which is depicted in (Figure 6(g)) proved the up-regulation of Bcl-2, Bcl-xl and Cyclin-D1 in miR-522-3p rescue HCC cells, while Bax abundance showed decrease in miR-522-3p mimics group (Figure 6(g)). Consequently, from the above discovery we could summarize that miR-522-3p promoted HCC proliferation via hindering cell apoptosis, and reversed the effect brought by DHRS4-AS1.

### 3.5. SOCS5 interacted with miR-522-3p and SOCS5 knockdown reversed the effects induced by miR-522-3p inhibitor

**Figure 7.**
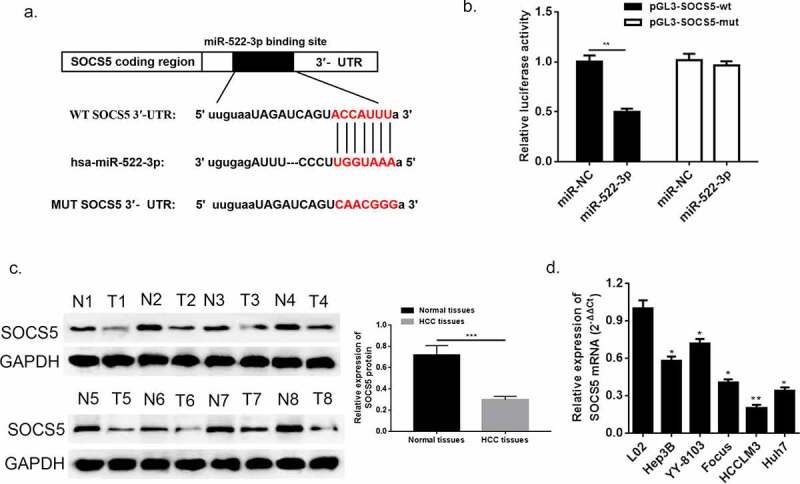
SOCS5 was identified as a target gene of miR-522-3p. (a) The bioinformatics prediction performed biding site. (b) The result of dual-luciferase assay. (c) Western blot and quantitative analysis showed that SOCS5 was down-regulated in HCC tissues. (d) QRT-PCR detected significant low expression of SOCS5 in HCC cell lines. N = 3, *p < 0.05, **p < 0.01

Suppressor cytokine signaling-5 (SOCS5) to date is recognized as a tumor suppressor in various types of cancer [25]. However, there is still little understanding about the pathological function of SOCS5 in HCC. In this study, we acquired a binding segment between miR-522-3p and SOCS5 from intersection from TargetScan, miRmap and miRanda (Figure 7(a)), which might suggest that SOCS5 might participate in regulating HCC progress via interacting with miR-522-3p. Corroboration of the bioinformatics prediction was conducted via dual-luciferase assay (Figure 7(b), p < 0.05), which confirmed the existence of direct interaction between SOCS5 and miR-522-3p. Next, the results of western blot showed that SOCS5 abundance was downregulated in HCC tissues when compared with adjacent normal tissues (Figure 7(c)). In addition, the mRNA expression of SOCS5 presented significant alleviation in HCC cell lines (Hep3B, YY-8103, Focus, HCCLM3 and Huh7) compared to normal hepatic cell line (L02) (Figure 7(d), *p < 0.05, **p < 0.01).

**Figure 8.**
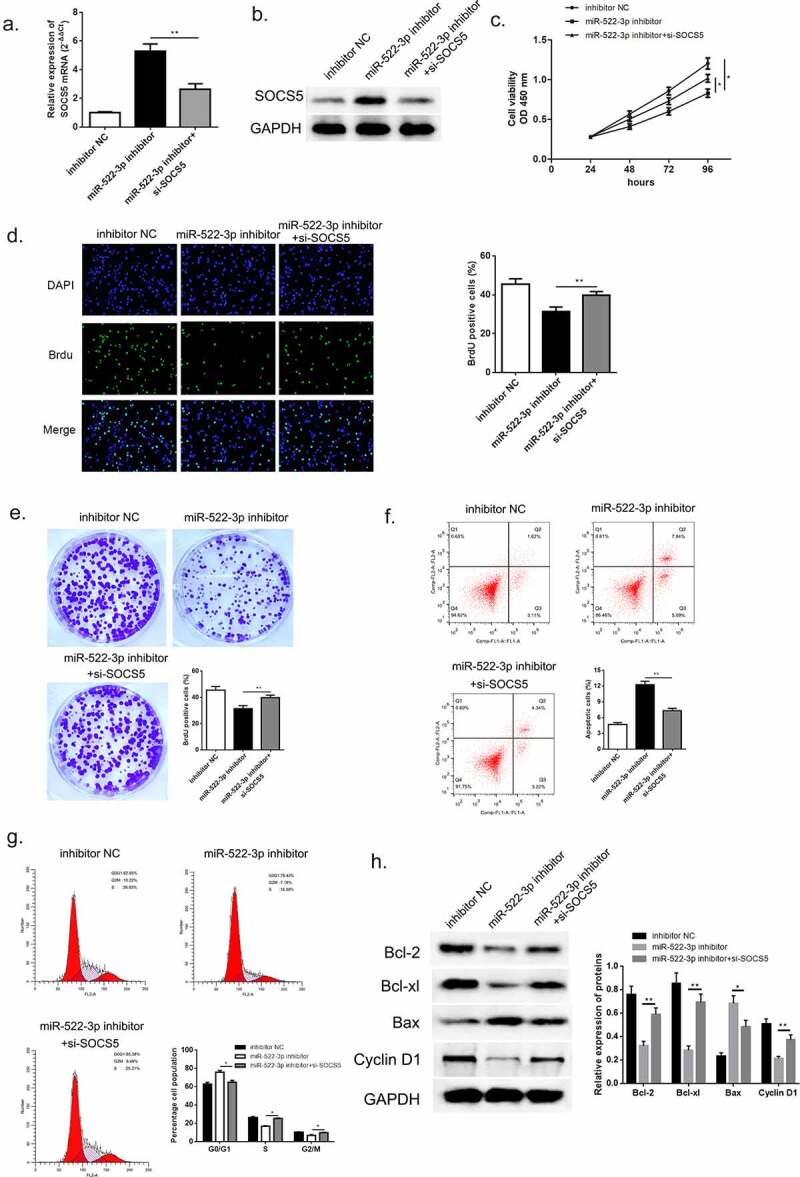
SOCS5 reversed the effects brought by miR-522-3p. (a) QRT-PCR and (b) western blot determined the expression level of SOCS5. (c) CCK-8 assay showed that SOCS5 alleviated cell growth in miR-522-3p inhibitor and si-SOCS5 co-treated group versus solo miR-522-3p inhibitor treated group. (d) The result of BrdU assay. (e) Colony-forming experiment showed that SOCS5 reversed the cell promoted effect brought by miR-522-3p. (f) Cell apoptosis rate and (g) cell cycle examined by flow cytometry. (h) The level and quantitative analysis of apoptosis related proteins’ cell cycle related protein’s abundance detected via western blot. N = 3, *p < 0.05, **p < 0.01

To further investigate the biological function of SOCS5 in HCC, we transfected si-SOCS5 and miR-522-3p inhibitor into HCCLM3 cells. QRT-PCR and western blot was used to validate the successful transfection of si-SOCS5; SOCS5 mRNA and protein was up-regulated when miR-522-3p was inhibited while the content dropped down when cells were given si-SOCS5 and miR-522-3p inhibitor simultaneously (Figure 8(a,b), p<0.01). Thereafter, the results of CCK-8, BrdU assay and colony-forming experiment exhibited that inhibition of miR-522-3p inhibitor would thereby restricted the proliferation of HCC cells; while knockdown of SOCS5 instead promoted cell growth of HCC, which suggested that down-regulation of SOCS5 was capable to reverse the HCC progress generated by miR-522-3p inhibitor (Figure 8(c–e)). The specific effects that exerted by SOCS5 were studied using flow cytometry and western blot. (Figures 8f) and 8 G manifested that inhibition of miR-522-3p inhibitor boosted cell apoptosis rate, yet silencing of SOCS5 significantly reversed this effect (p < 0.01). Also, cell cycle examination exhibited that down-regulation of SOCS5 lose the mighty ability to capture G0/G1 phase when compared with miR-522-3p inhibitor treated group (p < 0.05). Consistent with the consequences of flow cytometry, western blot showed that si-SOCS5 transfected HCC cells showed overexpression of Bcl-2, Bcl-xl and Cyclin-D1, and lower abundance of Bax compared to solo miR-522-3p inhibitor group (Figure 8(h)). From the above findings, we could draw a conclusion that SOCS5 participated in hindering the course of HCC in vitro via DHRS4-AS1/miR-522-3p axis.

## 4. Discussion

Recent decades, researchers have devoted much endeavor to disclose the biological function of lncRNAs [33]. It has been demonstrated that the abnormality of lncRNAs closely associated with the pathogeny of HCC [34,35], especially in a manner of mRNA-lncRNA-miRNA interaction [36]. This study was aimed to reveal the exact role of lncRNA DHRS4-AS1 in HCC and the potential underlying mechanism.

Primitive literature studies showed that lncRNA DHRS4-AS1 was considered to be tumor suppressed. For example, lncRNA DHRS4-AS1 functioned by preventing the proliferation and invasion, inhibiting the cell cycle progression and promoting the apoptosis of clear cell renal cell carcinoma [19]; lncRNA DHRS4-AS1 inhibited the stemness of non-small cell lung cancer Cells by Sponging miR-224-3p and Upregulating TP53 and TET1 [37]; and lncRNA DHRS4-AS1 was decreased in glioma and neuroblastoma [17,19], without further mechanism exploration. Interestingly, it was found that lncRNA DHRS4-AS1 was significantly reduced in HCC tissues by analyzing the public database TCGA yet its specific function and mechanism in HCC has to be investigated.

Firstly, we verified the significant reduction of lncRNA DHRS4-AS1 in 60 HCC tissues and HCC cell lines, compared to normal tissues and normal cell line, respectively. Additionally, the overall survival rate of HCC patients predicted using TCGA database exhibited that up-regulation of DHRS4-AS1 would help extend the survival rate. The expression of lncRNA DHRS4-AS1 was was significantly correlated to tumor size (*P* = 0.02) and TNM stage (*P* = 0.045) of HCC patients. All of these encouraged us to explore whether the level expression of DHRS4-AS1 would alter the HCC cell function.

Based on the results of CCK-8 assay, BrdU assay, colony-forming assay and flow cytometry, it was concluded that DHRS4-AS1 precluded HCC cell proliferation, promoted apoptosis and arrested cells in G0 phase *in vitro*. Furthermore, DHRS4-AS1 could serve a tumor suppressed function to slow down HCC tumor growth *in vivo*. On the basis of the findings mentioned before, we concluded that lncRNA DHRS4-AS1 was tumor suppressor in HCC. Yet the molecular mechanism of DHRS4-AS1 still needs lucubration. lncRNAs were usually considered as a ceRNA to affect the functional role of miRNAs to strengthen the functions [38].

Herein, we conducted bioinformatics prediction through miRanda database to find the potential targets of lncRNA DHRS4-AS1. Fortunately, it was found that DHRS4-AS1 could directly target miR-522-3p and overexpressed DHRS4-AS1 led to remarkable reduced miR-522-3p level. The binding interaction was also confirmed by dual-luciferase assay. The abundance of miR-522-3p in HCC tissues and cell lines were detected by qRT-PCR, and the results supported that miR-522-3p was overexpressed in HCC tissues and cell lines, that was in a negative-relationship with DHRS4-AS1. Notably, miR-522-3p has been reported to be significantly upregulated in diverse cancers, and promoted tumorigenesis as an oncogene, such as in colorectal cancer, osteosarcoma, glioblastoma and gastric cancer [39–42]. Our findings suggested that miR-522-3p rescued the effect of lncRNA DHRS4-AS1 on cell proliferation, apoptosis and cell cycle, indicating that DHRS4-AS1 acts as a sponge for miR-522-3p and DHRS4-AS1/miR-522-3p axis is involved in HCC progression.

Next, we also identified suppressor cytokine signaling-5 (SOCS5) was a negatively regulatory target of miR-522-3p using bioinformatics prediction and dual-luciferase assay. SOCS5 was also commonly found to be tumor-suppressed and significantly reduced in tumors, such as non-small cell lung cancer, pancreatic cancer and also liver cancer [43–45]. It was found that SOCS5 inhibition induced autophagy to impair metastasis in HCC cells via the PI3K/Akt/mTOR pathway [46]. Our results revealed that SOCS5 was positively correlated with DHRS4-AS1, and negatively correlated with miR-522-3p. SOCS5 knockdown could significantly rescue the effect of miR-522-3p inhibitor on HCC cells proliferation, apoptosis and cell cycle, indicating the interaction between miR-522-3p and SOCS5. It was generated a conclusion that SOCS5 also partook in mediating the development of HCC via DHRS4-AS1/miR-522-3p axis.

Additionally, the effect of lncRNA DHRS4-AS1/miR-522-3p/SOCS5 axis on the apoptosis related proteins and cell cycle related proteins was investigated, suggesting that this axis exert the function on HCC cells by altering the detected proteins expression.

## 5. Conclusions

Hepatocellular carcinoma (HCC) is a malignant tumor that seriously perils human health. In present study, we disclosed that lncRNA DHRS4-AS1 was capable to ameliorate the course of HCC by down-regulating miR-522-3p. Furthermore, SOCS5 was recognized as a negative target of miR-522-3p in HCC. Our findings concluded that DHRS4-AS1 improves HCC via miR-522-3p/SOCS5 axis.

## Data Availability

Research data could be available from the corresponding author.
